# Age Versus Immunity: Dietary Influences on Immunosenescence

**DOI:** 10.3390/jcm14238313

**Published:** 2025-11-22

**Authors:** Karolina Daniłowska, Natalia Picheta, Julia Piekarz, Dominika Żyła, Katarzyna Zych, Katarzyna Szklener, Sławomir Mańdziuk

**Affiliations:** 1 Student Academic Group, Department of Clinical Oncology and Chemotherapy, Medical University, 20-090 Lublin, Poland; karolina02812@gmail.com (K.D.); natalia.picheta2812@gmail.com (N.P.); piekarzjulia1@gmail.com (J.P.); dominika.zyla2211@gmail.com (D.Ż.); katarzyna.zych.98@gmail.com (K.Z.); 2 Department of Clinical Oncology and Chemotherapy, Medical University, 20-090 Lublin, Poland; slawomir.mandziuk@umlub.pl

**Keywords:** immunosenescence, microbiome, pesticides, vitamin D3, butyrate, omega-3 fatty acids, curcumin, functional and conventional foods

## Abstract

**Background**: Immunosenescence, or the age-related weakening of the immune system, leads to greater susceptibility to chronic diseases, infections, and metabolic disorders. This process involves changes in the number and function of lymphocytes, increased levels of inflammatory markers, and modifications to the gut microbiome. In recent years, increasing importance has been placed on diet—both functional, rich in bioactive ingredients, and conventional, often pesticide-laden—as a factor modulating immune system aging. **Methods**: This paper provides a literature review on the effects of dietary components on immunosenescence. Results from 2010 to 2025 and from clinical and experimental studies on substances such as curcumin, butyrate, vitamin D3, omega-3 fatty acids, and conventional products containing pesticides were analyzed. Their impact on the microbiome, intestinal barrier integrity, inflammatory biomarkers, and the overall immune response was considered. **Results**: Numerous beneficial effects of functional foods were identified. Curcumin increases microbiota diversity and lowers C-Reactive Protein (CRP) and Tumor Necrosis Factor α (TNF-α) levels. Butyrate supports remission in inflammatory bowel disease by reducing Interleukin (IL) 6 and TNF-α levels. Vitamin D3 lowers inflammatory markers and reduces calprotectin in inflammatory bowel disease. Omega-3 fatty acids modulate microbiome composition and improve lipid profiles. In contrast, conventional foods high in pesticides lead to dysbiosis, intestinal barrier damage, and increased pro-inflammatory cytokines. **Conclusions**: Diet is a key factor in modulating immunosenescence. Functional foods can support the microbiome and reduce chronic inflammation, while conventional foods may exacerbate the aging process of the immune system. Further clinical research is needed to develop dietary recommendations to support immunity in older adults.

## 1. Introduction

Aging of the human body is a multifaceted and dynamic process influenced by the interaction of genetic, environmental, epigenetic, and stochastic factors. Immunosenescence, or age-related weakening of immune system function, plays an important role in the functioning of the human body [1]. At the cellular level, it is manifested by reduced production of naive T and B lymphocytes, accumulation of memory and aging cells, and thymic involution and impaired cytokine regulation [2]. Consequently, it increases the risk of developing aging-related diseases such as neurodegenerative disorders, cancer, cardiovascular disease, autoimmune disease, and COVID-19, which can lead to organ failure and death [3]. One of the key aspects of immunosenescence is chronic low-grade inflammation, referred to as “inflammaging” [1]. In addition, the gut microbiome, changes in the thymus gland, diet, integrity of the mucosal barrier in the gut, physical activity, excessive stress, and sleep deprivation are found to have a role in advancing the aging process [4,5].

The integrity of the mucosal barrier, especially in the intestines, plays a key role in maintaining immune homeostasis. The intestinal barrier consists of a monolayer of epithelium that protects the body from the passage of pathogens and toxins from the intestinal lumen into the bloodstream. As we age, this barrier weakens, which can lead to increased intestinal permeability, known as “leaky gut”. The consequence is the entry of bacterial antigens into the systemic circulation, which stimulates chronic inflammation and enhances immunosenescence. However, this may be partially mitigated through targeted dietary interventions, as the composition and functionality of the gut microbiota in older adults appear to be strongly influenced by nutritional factors [6,7].

The intestinal microbiota, a complex community of microorganisms inhabiting the gastrointestinal tract, plays an important role in modulating the immune system. The proper composition of the microbiota supports intestinal barrier function and regulates the immune response. However, with age, changes in the composition of the microbiota are observed, such as a decrease in the number of probiotic bacteria of the Lactobacillus and Bifidobacterium genera, which can lead to dysbiosis [8]. Intestinal dysbiosis is associated with impaired intestinal barrier function, which promotes the penetration of pathogens and increases inflammation. In addition, the microbiota influences the production of secretory immunoglobulin A (sIgA), which is the first line of defense on the surface of mucous membranes, preventing the adhesion and penetration of pathogens. Reduced sIgA levels can lead to increased susceptibility to infection [9]. Whether gut dysbiosis and the worsening integrity of the mucosal barrier in the gut are a cause or an effect of aging remains to be determined [4].

In the context of aging populations worldwide, it is becoming important to look for strategies to support immune function in old age. One of the key factors influencing the health of the immune system is diet [5]. Conventional foods provide essential nutrients, such as vitamins and minerals, which are the foundation for proper functioning of the body. However, in recent years there has been growing interest in functional foods, which offer additional health benefits in addition to their basic nutritional value [10].

Functional foods are products enriched with bioactive ingredients, such as probiotics, prebiotics, omega-3 fatty acids, antioxidants, or herbs, which are designed to support specific bodily functions, including the immune system [11]. Dietary sources of probiotics include fermented foods such as yogurt and kefir. Prebiotics, such as inulin and fructooligosaccharides, provide nourishment for beneficial intestinal microflora, promoting its development and activity. Food rich in prebiotics are chicory, garlic, onions, and bananas, among others. Regular consumption of prebiotics can lead to a lower risk of gastrointestinal infections and support overall immunity [12,13].

Another important component is omega-3 fatty acids, present in marine fish, flaxseed, or walnuts. They have been shown to have anti-inflammatory effects and to modulate the immune response, which may be particularly beneficial in the context of immune aging [1]. Aging on the other hand, may be perceived as a consequence of chronic oxidative stress, resulting from an excess of free radicals, leading to progressive accumulation of biomolecular damage. In this context, it is also important to consume foods rich in antioxidants, such as vitamins A, C, and E, which neutralize free radicals and protect cells from oxidative stress. Vegetables and fruits, such as carrots, peppers, citrus, and berries, are excellent sources of these compounds [14].

In an era of rapid aging of populations around the world, the topic of immunosenescence is gaining particular importance. Increasing life expectancy poses new challenges for medicine and dietetics in maintaining health and immunity in the elderly. Since immunosenescence disrupts the balance between pro- and anti-inflammatory mechanisms, a shift toward pro-inflammatory activity may accelerate the onset and progression of aging-related diseases. As a result, there is growing interest in the role of diet and functional foods as a potential strategy for supporting the immune system naturally. In this review, the authors focused on the effects of functional and conventional diets on the aging of the immune system. Special attention was paid to the effects of substances such as vitamin D3, butyrate, omega-3 fatty acids, curcumin, and pesticides.

## 2. Materials and Methods

The review was conducted in accordance with the principles of a systematic literature review. Publications available in the PubMed, Scopus, and Web of Science databases, covering the period from 2010 to 2025, were analyzed using the following keywords: immunosenescence, microbiome, pesticides, vitamin D3, butyrate, omega-3 fatty acids, curcumin, and functional and conventional foods. English-language papers published in peer-reviewed scientific journals were included. Ultimately, 84 publications that met the qualitative and substantive criteria were included in the narrative synthesis.

Inclusion criteria were as follows: clinical, cohort, experimental, and review studies on immunosenescence, the microbiome, or diet (functional and conventional); publications assessing the effects of dietary components (curcumin, butyrate, vitamin D3, omega-3 fatty acids, probiotics, prebiotics) on the immune system and inflammatory processes; and studies focusing on adult populations, including older adults.

Exclusion criteria were as follows: articles in languages other than English; popular science papers or papers that did not meet the review criteria; studies involving only animal models without any human references; and publications with incomplete data or unavailable full text.

The publication selection process was conducted independently by two authors, and any discrepancies were resolved through discussion.

## 3. Immunosenescence and Inflammation

The immune system gradually weakens with age, leading to an imbalance between pro-inflammatory and anti-inflammatory responses. Those changes, referred to as “immunosenescence”, are processes that influence both natural and acquired immunity [15]. They promote the maintenance of low levels of systemic inflammatory state, which increases the risk of developing chronic diseases and disability. As a result, susceptibility to cardiovascular disease, cognitive decline, metabolic disease, debilitation, and the risk of death increases [16,17]. The increase in inflammation that occurs with age can also lead to failures in the treatment of certain chronic diseases, cancers, or wound management [18].

The literature identifies changes in CD (Cluster of Differentiation) 4/CD8 ratio (<1), increased Interleukin (IL) 6, high C-Reactive Protein (CRP) levels, neutrophilia, and high tumor necrosis factor α (TNF-α) levels as key biomarkers of the aging immune system [18,19]. Van Hoi et al., who analyzed 44 cross-sectional studies related to immunosenescence biomarkers among older individuals free from active conditions that could influence immune parameters, found that frailty syndrome was most frequently associated with IL-6 [19]. One of the main functions of IL-6 is to suppress the inflammatory response by inhibiting the production of TNF-α and IL-1β. Consequently, IL-6 can simultaneously regulate both pro-inflammatory and anti-inflammatory activity, contributing to both intensification and suppression of the acute phase immunological response [20]. With age, there is a decrease in the number of CD8+ T cells and an increase in the CD4/CD8 ratio. In addition, there is a significant increase in the number of natural killer (NK) cells. The number of CD4+ T lymphocytes appears to be less susceptible to the aging process compared to CD8+ T lymphocytes and other immune cell subgroups [21].

Although cellular aging is crucial for tissue homeostasis, its disruption has been linked to diseases such as cancer, premature aging, and age-related diseases. DNA damage plays a role in the aging process, but the exact mechanism of this phenomenon remains unknown [22]. Yang et al. suggest an immunosenescence-related biochemical response regulated by cyclic guanosine monophosphate-adenosine monophosphate synthase (cGAS) deoxyribonucleic acid (DNA) sensor, which is crucial for the induction of cellular aging. Deletion of cGAS accelerates spontaneous immortality in mouse embryonic fibroblasts. The absence of cGAS prevents the expression of cell aging-specific inflammatory genes in response to DNA damage [23]. A role in reducing DNA damage is also speculated for mitochondrial telomerase reverse transcriptase (TERT). Increased levels of TERT contribute to a reduction in the area of myocardial damage after ischemic incidents, indicating its potential as a therapeutic strategy in protecting cellular DNA by contributing to the inhibition of adverse cellular aging processes [24].

## 4. Immunosenescence and Changes in the Mucosal Barrier and Microbiome

Human intestines are colonized by many commensal organisms, viruses, bacteria, and fungi. The largest number and greatest diversity of microorganisms are found in the colon, with 1010–1012 organisms per 1 g of lumen content. It is well known that the microbiota has a significant impact on the physiological processes of the host, including immunity. Some of the organisms that inhabit the intestines have the ability to produce immunoglobulin A (IgA) and develop T-effector cells (Th1/Th17) or regulatory T cells (Treg). *Bacteroides fragilis*, through activation of the Toll-like receptor (TRL2), is indirectly involved in the development of Foxp3+ Treg cells and the secretion of IL-10 as a result of the expression of the anti-inflammatory polysaccharide A (PSA) [25]. TRLs are a group of pattern recognition receptors that are capable of recognizing bacterial, viral, and parasitic ligands. Their activation induces nuclear transcription factor-kappa B (NF-kB), which is involved in the production of inflammatory cytokines [26]. The microbiome undergoes constant changes throughout life. The final individual “profile” is established around age 2. In the digestive tract, beta diversity or diversity among individuals, is greatest in children, while alpha diversity, or diversity within an individual, increases in adulthood and remains constant until old age [27]. In older people, alpha diversity begins to decline due to changes in the numbers of some bacterial species. The composition of the microbiome changes, which affects inflammatory processes. The causes of changes in the composition of the microbiome include increased use of antibiotics in older people, reduced gastric motility, and changes in diet and lifestyle [27].

A number of studies conducted by researchers have discussed the impact of the abundance of specific bacterial species on the diseases that older people develop. As people age, *Oscillibacter* levels decrease, while *Bacteroides* levels increase, which correlates with increased CRP levels. In the study, lower levels of *Ruminococcus* and *Prevotella* bacteria coincided with increased levels of IL-6 [28]. In atherosclerosis-related cardiovascular diseases, increased numbers of *Proteobacteria* and decreased numbers of *Bacteroides*, *Prevotellam,* and *Faecalibacterium* contributed to the occurrence of ischemic strokes of large arteries [29]. In addition, the number of *Eubacterium ventriosum* bacteria increases, which correlates with a decreased number of lymphocytes. The decrease in the number of *Bifidobacterium* bacteria, which presumably play a significant role in protecting the lipid barrier, is associated with an increase in high-sensitivity C-Reactive Protein (hsCRP) concentration [29].

Changes in the composition of the microbiome have also been associated with a higher incidence of neurodegenerative diseases such as Parkinson’s and Alzheimer’s.

Under physiological conditions, the mucus layer forms a shield that limits the adhesion of pathogens to the underlying epithelium. The most prominent change in the aging mucosa is a decrease in the secretion of IgA, which is crucial in protecting the host’s body from pathogens [30]. In addition, studies in mice have confirmed that there is a progressive decrease in the size of Peyer’s tufts with age. This appears to be due to a decrease in lymphotoxin β (LTβ) signals, which play a key role in maintaining Peyer’s tufts [30].

Equally important components that provide immunity are endothelial lymphocytes and Paneth cells in the small intestine. Paneth cells exert their antimicrobial effect by secreting the immunomodulatory lysozyme. Also playing an important role in intestinal immunity is IL-22, which is involved in epithelial regeneration and the release of antimicrobial peptides [31]. In elderly people, inflammatory processes and concentrations of free radicals (ROSs) increase, resulting in damage to the intestinal barrier and slowing regenerative processes. Particularly susceptible to destruction by inflammatory factors are tight junction proteins, zonulin and claudin, resulting in unsealing and increased permeability of the intestinal barrier [32]. This leaves the elderly vulnerable to the occurrence of many infections. In addition, the production of mucin, which is the main component of the intestinal mucosa, decreases with age, making it thinner and easier for microorganisms to penetrate. Mucin is also a source of nutrients for bacterial strains such as *Clostridiaceae*, *Akkermansiaceae*, *Bifidobacteriaceae*, and *Bacteroidaceae*, the number of which decreases with age [33].

## 5. The Impact of Functional Foods on the Microbiome and Components of the Immune System

With aging, the body undergoes various changes, including microbiome dysbiosis and alterations in components of the immune system [34]. However, these changes can be counteracted, for example, through the use of certain functional foods. These are foods defined as products that exert targeted physiological effects, promoting health and aiding in disease prevention [35]. This section will focus on the beneficial effects of *Curcuma longa*, butyrate, vitamin D3, and omega-3 fatty acids on immunosenescence.

### 5.1. Curcuma longa

*Curcuma longa* has many pharmacological properties ranging from anti-inflammatory, antioxidant, antimicrobial, anticancer, and anti-aging effects [36]. It is estimated that the global market for turmeric in 2016 was half a billion dollars [37]. Turmeric has been recognized by the Food and Drug Administration (FDA) as Generally Recognized as Safe (GRAS), and the daily single well-tolerated dose of curcuminoids can reache up to 12 g/day [36,38]. The most important substances in the plant are curcumin (60–70%), demethoxycurcumin (20–27%), and bisdemethoxycurcumin (10–15%) [39].

Curcumin is credited with numerous mechanisms for reducing inflammation. It binds to Toll-like receptors (TLRs), thereby regulating the expression of proteins such as NF-κB, mitogen-activated protein kinase (MAPK), or activation protein 1 (AP-1), contributing to a decrease in inflammatory mediators [40]. In addition, by acting on peroxisome proliferator-activated receptor gamma (PPARγ), it contributes to a decrease in NF-κB, which stimulates the production of pro-inflammatory cytokines, including IL-1, IL-6, IL-8, or TNF-α [41,42]. Aging is characterized by a low level of inflammation associated with immunosenescence, resulting from chronic activation of NF-κB signaling. Furthermore, NF-κB, interacting with the mammalian target of rapamycin (mTOR), another age-related pathway, creates a network that regulates cellular aging. The mTOR pathway is dysregulated in aging immune cells. Consequently, its persistent activation leads to a shortened lifespan. T cells lose CD28 expression and acquire a senescent phenotype. Interestingly, mTOR activation leads to increased T-cell differentiation toward short-lived effector cells rather than memory cells, reducing immunological memory in older individuals. Dietary curcumin reduces the production of the aforementioned pro-inflammatory cytokines, thus inhibiting the age-associated secretory phenotype (SASP). There is no dysregulation of nutrient-sensing, cellular stress, and inflammation pathways [2]. In addition, it modifies the microbiome by increasing the number of bacterial strains and promoting beneficial species. Many studies performed in animal models and increasingly in humans confirm the positive effects of curcumin on the immune system [43].

A study evaluating the effects of turmeric on the gut microbiome involved thirty participants, divided into three groups of ten. Each group was given different pills: a placebo, turmeric 1000 mg + 1.25 mg of elderberry piperine alkaloid extract (BioPerine), and curcumin 1000 mg + 1.25 mg Bioperine. Each patient was advised to take three tablets twice daily. Analysis of the number of bacterial species before and after treatment was performed. It was concluded that in the placebo group, the number of strains decreased from 175 to 149 (by 15%), while the group taking turmeric increased from 156 to 167 (by 7%). On the other hand, the biggest change was seen in patients taking curcumin, where the number of strains increased from 127 to 215, an average increase of 69%. The results indicate that curcumin has an effect on expanding the microbiome [44].

Fifty-six men were evaluated and divided into two equal groups: a control group taking a placebo and a study group taking 80 mg of nanomicelle curcumin daily, in a randomized, double-blind, placebo-controlled clinical trial. Changes in CRP and TNF-α concentrations were analyzed, and it was inferred that there was a significant decrease in the research group. The average change in CRP in the control group was −0.1 ± 1.84, whereas in the study group it was −1.64 ± 1.5. Similarly, TNF-α levels were −0.12 ± 1.51 in the control group and −1.5 ± 1.5 in the study group [45].

A triple-blind, placebo-controlled, randomized clinical trial involved forty patients divided into two groups, taking either a placebo or curcuminoid nanomicelles (Sinacurcumin^®^ 40 mg). Tablets were taken four times a day. Cytokine levels were examined on days 0, 7, and 14. The results showed that Sinacurcumin^®^ significantly decreased interferon gamma (IFN-γ) and transforming growth factor beta (TGF-β) levels and increased IL-4 and IL-17, which suggests that curcumin has anti-inflammatory properties [46].

### 5.2. Butyrate

Butyrate is classified as a short-chain fatty acid (SCFA). It is produced in the gastrointestinal tract through bacterial fermentation of dietary fiber [12]. SCFAs contribute to the function of the epithelial, nervous, immune, and vascular systems. In addition, they are characterized by anti-apoptotic and anti-tumor effects through histone deacetylase (HDAC) inhibition [47].

Butyrate has numerous mechanisms responsible for its anti-inflammatory effects. It activates numerous G protein-coupled receptors (GPCRs). Butyrate has a high affinity for G protein-coupled receptor (GPR) 43, which is highly concentrated in immune cells. In addition, through stimulation of GPR109A, there is a differentiation of regulatory T cells and an increase in IL-10 and IL-18 levels [48]. Moreover, it is attributed to inhibit the expression of genes responsible for the production of pro-inflammatory NF-κB, TLR2, vascular endothelial growth factor A (VEGFA), IFN-γ, IL-1β, IL-6, and IL-12 [49]. TLR2 is an innate immune sensor. Butyrate inhibits its sustained activation by pathogen-associated molecular patterns (PAMPs) and damage-associated molecular patterns (DAMPs), limiting inflammatory responses. Older age is associated with increased accumulation of endogenous DAMPs and, consequently, the occurrence of chronic low-grade inflammation [2]. Another mechanism of butyrate, similar to curcumin, is the inhibition of mammalian target of rapamycin kinase (mTOR) and PPAR-γ [50].

A study used nucleus pulposus cells isolated from seven patients after lumbar injury, to which butyrate was added after a 24 h incubation at doses of 0, 0.1, 0.2, 0.5, 1, 2, and 5 mM. In addition, IL-1β at a dose of 10 ng/mL, was added to the cells to establish an inflammatory model. Analysis of the results showed that IL-6 and TNF-α concentrations were reduced. TNF-α concentration in the group with butyrate at a dose of 0 mM was 120 pg/mL, whereas at a dose of 2 mM it was only 25 pg/mL. Similarly, Il-6 levels were 280 pg/mL and 175 pg/mL, respectively [51].

Another study included forty-two patients with ulcerative colitis (UC) in remission who were randomly divided into two groups. Their standard mesalamine-based therapy (dose 2400 mg/d) was supplemented with either a placebo or sodium butyrate (BLM) at a dose of 1000 mg. Analysis of the results showed that remission was maintained in 83.3% of patients taking BLM and 47.6% of patients in the control group. In addition, subjective improvement in well-being after six months was assessed, where it was achieved by 61.1% of patients taking BLM and 14.3% taking placebo [52].

Forty-nine patients with inflammatory bowel disease (IBD) were included in the study, including nineteen with Crohn’s disease (CD) and nineteen with UC. The patients were randomly divided into two groups, with one taking placebo and the other taking sodium butyrate 1800 mg/d (Butyrose^®^). In addition, 18 healthy volunteers (HVs) were also recruited. This made it possible to conclude that microbiota richness in IBD patients was much less developed than in HV. Analysis of the results showed that UC patients taking butyrate had a significant increase in bacteria from the Lachnospiraceae family, while CD patients were noted to have an increase in strains from the *Butyricicoccus* genus [53]. Both bacterial strains are credited with anti-inflammatory effects [54].

### 5.3. Vitamin D3

Vitamin D3 is classified as a fat-soluble compound. It is produced in the human body under UVB from 7-dehydrocholesterol (7-DHC) and is then converted in the liver to the circulating form 25-hydroxyvitamin D3 [25(OH)D3]. This form reaches the kidneys and is converted to the most active form, 1,25-dihydroxyvitamin D3 [1,25(OH)2D3], through cytochrome p450 family 27 subfamily B member 1 (CYP27B1) [55]. It is responsible for maintaining normal calcium–phosphate homeostasis and controlling bone turnover [56]. In addition, it has been attributed with many additional properties, including anti-inflammatory, antioxidant, DNA repair, anticancer, antimicrobial, and immunomodulatory effects [57,58]. There are multiple mechanisms responsible for the anti-inflammatory properties of vitamin D3. By affecting T-cell differentiation, it causes the conversion of pro-inflammatory to anti-inflammatory responses. This occurs through inhibition of Th1 and a decrease in IL-2 and IFN-γ, and stimulation of Th2 with an increase in IL-4. In addition, it is essential for the proper functioning and differentiation of Tregs, which reduces the likelihood of autoimmune diseases [59]. In addition, it is credited with inhibiting the synthesis and secretion of IL-6, TNF-α, and IL-1β. Another of vitamin D3’s actions is its effect on Th17 cells, through which the expression of IL-17, IL-22, TNF-α, IFN γ, and the chemokine receptor CCR6 is inhibited. What is more, in a population of healthy older adults, the serum concentration of 25-hydroxyitamin D was found to correlate with the cytolytic activity of NK cells [60]. Another study recruited 50 men who were orally administered a single dose of 80,000 IU of vitamin D3. After analysis of pro-inflammatory markers, it was inferred that the vitamin has anti-inflammatory properties [61]. The effect of vitamin D3 on the concentrations of selected pro-inflammatory markers is presented in Table 1.

The study included 25 patients: 8 with active UC, 9 with inactive UC, and 8 controls without IBD. They were given 40,000 UI of vitamin D3 per week for eight weeks. Fecal analysis showed that there was a significant decrease in calprotectin levels after vitamin D supplementation in the group of patients with active UC (median 257 to 111). In addition, changes in the gut microbiota were analyzed. A significant decrease in Ruminococcus gnavus was observed in all patients who supplemented with vitamin D3 [62]. Significant effect on the development of inflammation in the gut is attributed to this bacterium, as it activates the production of pro-inflammatory cytokines [63].

### 5.4. Omega-3

Omega-3 fatty acids may have prebiotic functions. Even a low dose of 500 mg of eicosapentaenoic acid (EPA) + docosahexanoic acid (DHA) daily for six weeks can induce small but consistent changes in the composition of the gut microbiota (e.g., an increase in *Coprococcus* and *Bacteroides*) and increase the production of beneficial metabolites such as short-chain and branched-chain short-chain fatty acids [64,65]. Fish oil supplementation effectively lowers levels of key diabetes indicators (HbA1c, HOMA-IR) and improves lipid profile. Omega-3s from fish oil favorably alter the composition of the intestinal microbiota, reducing the abundance of potentially pathogenic bacteria (*Desulfobacterota*, *Colidextribacter*, *Ralstonia*, *Klebsiella*) and increasing the abundance of beneficial species (*Limosilactobacillus*, *Lactobacillus*, *Haemophilus*). Similar changes are observed in the intestinal fungal population. Nevertheless, the overall quality of evidence requires careful consideration. Although the study applied a rigorous double-blind, placebo-controlled design, its relatively small sample size and potential influence of uncontrolled confounding factors limit the strength and generalizability of the conclusions [66].

The study of Lu et al. analyzed the effects of fish oil (FO) supplementation on triglyceride (TG) levels, serum lipidomics, and gut microbiota in 309 Chinese patients with type 2 diabetes (T2D) and hypertriglyceridemia (HTG). FO supplementation significantly reduced fasting triglyceride levels in the FO group compared to the placebo group but had a limited effect on the gut microbiota [67].

This study of Hornero-Ramirez et al. provides evidence that an 8-week intervention with a multifunctional cereal product can benefit gut health and some aspects of metabolism in people, especially in people at cardiometabolic risk. MF promoted the growth of beneficial bacterial species such as *Bacteroides ovatus*, *Bacteroides uniformis,* and *Agathobaculum butyriciproducens*, which is associated with the observed anti-inflammatory effects [68]. Table 2 presents the key effects, detailed changes, and study details for omega-3 and the multifunctional cereal.

## 6. The Impact of Conventional Food on the Microbiome, Mucosal Barrier Integrity, and Inflammation in the Elderly

Conventional food production aims to increase the quantity of products while minimizing the costs of their production. To achieve this goal, pesticides, artificial fertilizers, and growth stimulants must be used [69]. In connection with this, increased consumption of pesticides has been observed in the world [70]. Moreover, this is also caused by genetically modified crops, which provide tolerance to synthetic pesticides [71].

Pesticides constitute a broad group of substances, both synthetic and natural. They are divided into insecticides, fungicides, and herbicides [72]. To date, studies confirm the toxic effects of pesticides on the human body, including carcinogenicity, neurotoxicity, endocrine and developmental disruption, and metabolic toxicity [73,74,75,76,77]. Humans are mainly exposed to them through the consumption of contaminated food [78]. As a result, they are classified as substances that disrupt the intestinal microbiota, compromise epithelial barrier integrity, and consequently alter inflammatory responses [70,71]. The presence of pesticides in the intestinal lumen causes a decrease in the expression of intestinal bacterial DNA, leading to dysbiosis. Pesticides affect the intestinal epithelial barrier through mechanisms dependent on the activity of ROS. They reduce the expression of molecules forming tight junctions (TJs), leading to increased barrier permeability. Additionally, they induce a cytotoxic effect by changing the integrity and function of the cell layer, affecting alkaline phosphatase activity, antioxidant enzyme activity, lipid peroxidation, Akt activation, and finally apoptosis. Moreover, as a result of inducing oxidative stress, they reduce the height of the jejunal villi and the expression of messenger ribonucleic acid (mRNA) of TJ protein genes. There is an increase in pro-inflammatory cytokines such as IL-1β, IL-6, TNF-α, NF-kB, and in the expression of cyclooxygenase (COX) 2 in the intestinal mucosa. Persistent exposure to pesticides is an important factor in the development of an inflammatory response. In their presence, the NLR family pyrin domain-containing 3 (NLRP3) inflammasome is activated. Its caspase-1 cleaves pro-IL-1β and pro-IL-18 into their mature forms, which are essential for the immune response [71]. Interestingly, up to 70% of the human body’s lymphocyte population is located in the gastrointestinal tract. They play the role of the first line of immune defense by preventing inflammation. These include natural intraepithelial lymphocytes (IELs), CD8αα+CD4+IEL, and TCRαβ-induced IEL [79]. Most mechanisms of pesticide action on intestinal barrier integrity have been identified through studies performed on animal models. However, there are also publications of studies involving humans in the literature.

In one study, 27 people poisoned by oral ingestion of pesticides were evaluated. Levels of IFN γ, IL-1β, IL-6, and TNF-α were measured. The types of pesticides ingested were also determined, including glufosinate (n = 6), glyphosate (n = 8), organophosphate (n = 4), pyrethroid (n = 2), and others (n = 7). IFN-γ levels were 2.78 ± 8.03 pg/mL, IL-1β 2.62 ± 2.03 pg/mL, IL-6 44.58 ± 80.16 pg/mL, and TNF-α 11.80 ± 15.60 pg/mL. Furthermore, the overall mortality rate was 11.1% (3/27). In addition, the level of IL-1β and TNF-α in the death group was significantly higher compared to the survivors [80]. Exposure of the gastrointestinal tract to ingested pesticides leads to inflammation.

Another research included 14 people exposed to pesticides and 12 people without documented exposure to pesticides (environmental or dietary). An increase in IL-6 (*p* < 0.05) and IL-12 (*p* < 0.01), and a decrease in IL-10 (*p* < 0.01) were observed in the exposed group. An increase in TNF-γ was also noted in the exposed group, but this was without statistical significance (*p* = 0781). Additionally, in the above group, a positive correlation was shown between CD4+ lymphocytes levels and IL-10 (*p* < 0.05), as well as between CD4+ lymphocytes levels and IL-6 (*p* < 0.05) [81].

Another study focused on 2847 participants to examine the association between pesticide exposure and inflammation, using CRP and the CRP-to-serum albumin ratio (CAR) as indicators. Participants were divided into three groups based on pesticide exposure (low, medium, and high). Significant positive associations with CRP were noted for medium- (β = 0.191, 95% CI: 0.084, 0.299, *p* = 0.001) and high- (β = 0.384, 95% CI: 0.280, 0.488, *p* < 0.001) exposure groups compared to the low-exposure group. Similarly, positive correlations with CAR were detected in the moderate- (β = 0.198, 95% CI: 0.087, 0.310, *p* = 0.001) and high- (β = 0.400, 95% CI: 0.295, 0.505, *p* < 0.001) exposure groups [82].

In another study, 60 people were divided into 2 groups, one of which included 47 people exposed to pesticides and the other included 13 healthy people. The inflammatory response to pesticides was examined in the exposed people. A significant increase in two immune markers was found: endothelin (*p* = 0.038) and monocyte chemotactic protein 1 (MCP-1) (0.04). Additionally, a significant difference in these markers was shown in comparison to the healthy group (*p* < 0.05), where the levels of markers were lower [83]. Table 3 presents the key effects, detailed changes, and study details of the pesticides.

## 7. Discussion

Immunosenescence is the weakening of immune system function. Its components include decreased production of naive T and B lymphocytes, thymic involution, cytokine dysregulation, and the accumulation of memory and aged cells. As a consequence of these changes, there is an increased risk of developing many age-related diseases.

As the immune system ages, an imbalance occurs between pro-inflammatory and anti-inflammatory processes. This is mainly due to changes in the CD4/CD8 ratio, increased levels of IL-6, elevated CRP, neutrophilia, and high levels of TNF-α. Particular attention is drawn to the change in IL-6 levels, which can exert both pro-inflammatory and anti-inflammatory effects. It is also important to note that CD4+ lymphocytes appear to be less susceptible to aging-related processes.

The human microbiome is an important component of immunosenescence, and changes in specific bacterial strains have a significant impact on inflammatory processes. The bacteria inhabiting the gut influence components of the immune system, such as IgA and effector and regulatory T cells. Alterations in the microbiome are associated with neurodegenerative and ischemic brain diseases. As the body ages, changes occur in the mucosal membrane, primarily a decrease in mucin production, which serves as a barrier against microorganisms and as a source of nutrients for gut bacteria.

Functional food includes products aimed at supporting the immune system by being enriched with bioactive components. Turmeric, as a functional food, reduces inflammation. Studies have shown that curcumin has a positive effect on expanding the microbiome by up to 69%, significantly decreases the levels of CRP, TNF-α, IFN-γ, and TGF-β, and increases the levels of IL-4 and IL-17. Butyrate, on the other hand, has demonstrated a significant effect in reducing IL-6 and TNF-α levels in studies. It also showed an impact on extending remission in patients with UC by 35.7% compared to the control group and improved the well-being of 46.8% of patients compared to those receiving a placebo. In patients with IBD, it positively influenced the growth of anti-inflammatory bacterial strains such as *Lachnospiraceae* in UC patients and *Butyricicoccus* in those with CD. Vitamin D3 has been shown in studies to have a significant effect on reducing inflammatory markers. Additionally, in patients with ulcerative colitis (UC) who received 40,000 IU of vitamin D3 per week for eight weeks, there was a significant reduction in calprotectin levels. Researchers also emphasize the impact of vitamin D3 on lowering the levels of pro-inflammatory cytokines.

Conventional food is the opposite of functional food. Mass-produced, it is often rich in pesticides, artificial fertilizers, and growth stimulators. Pesticides are not only carcinogenic and neurotoxic but also cause endocrine disorders, dysbiosis, and developmental and metabolic disturbances. They also lead to an increase in pro-inflammatory cytokines such as IFN-γ, IL-1β, IL-6, and TNF-α, elevated inflammatory markers, and immune markers such as endothelin and MCP.

**Figure 1:**
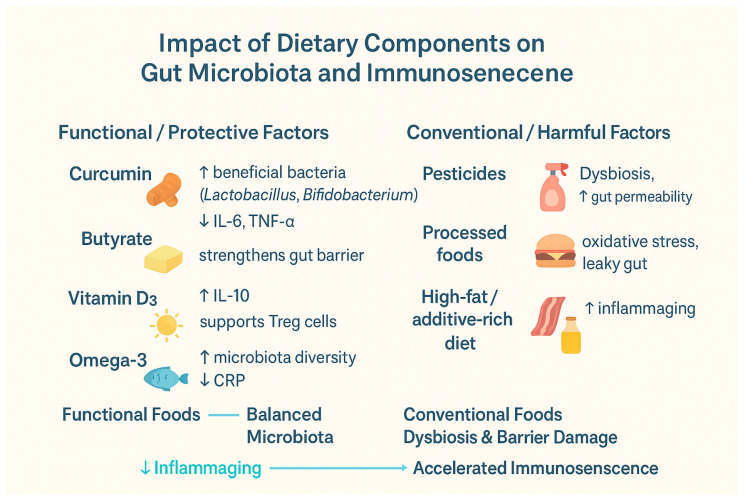
Summarizing the impact of various dietary components on the aging immune system and microbiota: IL-6—Interleukin 6, IL-10—Interleukin 10, TNF-α—Tumor Necrosis Factor Alpha, and CRP—C-Reactive Protein, Treg—Regulatory T Cells [44,45,46,51,52,53,61,62,67,68,82,83]. ↑—increase; ↓—decrease.

Table 4 and Figure 1 summarize the impact of various dietary components on the aging immune system and microbiota.

It is also believed that greater attention should be directed toward implementing dietary recommendations in older adults. This can be achieved through the integration of nutritional prevention programs in senior care facilities and by educating patients and caregivers. In practice, the program may include the following: (1) assessment of nutritional status (e.g., micronutrient deficiencies) at the beginning of the program, (2) introduction of dedicated meals or supplementation in senior care facilities, (3) training for caregivers and seniors on how to choose natural products rich in vitamins D and E, zinc, selenium, omega-3 fatty acids, and other nutrients, and (4) monitoring of effects (e.g., inflammation markers or lymphocyte subpopulation counts). This type of public health strategy could represent a relatively low-cost approach with broad beneficial implications in aging societies [84].

## 8. Conclusions

Immunosenescence represents an important direction for future research, as the current society’s lifespan is significantly increasing, creating a need to further explore this issue. A greater number of studies will make it possible to develop dietary guidelines or protocols aimed at maintaining a diverse and healthy microbiome for as long as possible. This could help prevent the development of many diseases and slow the progression of existing conditions. Additionally, conducting more research on changes in the components of the immune system will support the development of future strategies to prevent or mitigate these changes.

## Figures and Tables

**Table 1:** Concentrations of pro-inflammatory markers before intake, on day 1, and on day 30 after vitamin D3 intake [61].

Concentration of Substances (ng/L)	Baseline	Day 1	Day 30
IL-6	400	340	130
IL-8	500	440	200
TNF-α	150	140	60

IL-6—Interleukin-6; IL-8—Interleukin 8; TNF-α—Tumor Necrosis Factor α.

**Table 2:** Key effects, detailed changes, and study details for omega-3 and the multifunctional cereal [67,68].

Substance	Key Effects	Detailed Changes	Study Details
Omega-3 Fatty Acids [67]	FO supplementation significantly reduced fasting TG levels.	Low dose (500 mg EPA+DHA daily for six weeks) can increase *Coprococcus*, *Bacteroides*, and SCFA production. Reduction in HbA1c and HOMA-IR. Reduction in *Desulfobacterota*, *Colidextribacter*, and *Ralstonia*, *Klebsiella*; increase in *Limosilactobacillus*, *Lactobacillus*, and *Haemophilus*. Limited effect on gut microbiota.	309 Chinese patients with T2D and HTG. FO supplementation vs. placebo.
Multifunctional Cereal Product [68]	Benefits gut health and some aspects of metabolism, especially in people at cardiometabolic risk. Anti-inflammatory effects.	Promoted growth of beneficial bacterial species: *Bacteroides ovatus*, *Bacteroides uniformis*, and *Agathobaculum butyriciproducens*.	8-week intervention.

FO—fish oil; TG—triglyceride; EPA—eicosapentaenoic acid, DHA—docosahexanoic acid; HbA1c—hemoglobin A1c; HOMA-IR—homeostatic model assessment of insulin resistance; T2D—type 2 diabetes; HTG—hypertriglyceridemia.

**Table 3:** Key effects, detailed changes, and study details of the pesticides.

Substance	Key Effects	Detailed Changes	Study Details
Pesticides [80]	Assessment of the impact of acute pesticide poisoning on cytokine levels and mortality.	Assessment of the impact of acute pesticide poisoning on cytokine levels and mortality.	27 individuals poisoned by oral ingestion of pesticides (glufosinate, glyphosate, organophosphate, pyrethroids, and others). Overall mortality 11.1%.
Pesticides [81]	Comparison of cytokine levels in individuals exposed and unexposed to pesticides.	In the exposed group: increase in IL-6 and IL-12 and decrease in IL-10. Increase in TNF-γ (not statistically significant, *p* = 0.0781).	14 individuals exposed to pesticides, 12 individuals without documented exposure.
Pesticides [82]	Study of the association between pesticide exposure and inflammation indicators (CAR).	Significant positive associations with CRP for medium and high exposures. Similar positive correlation with CAR for medium and high exposures.	2847 participants were divided into low-, medium-, and high-pesticide exposure groups.
Pesticides [83]	Study of the inflammatory response (immune markers) in individuals exposed to pesticides.	Significant increase in endothelin and monocyte chemotactic protein in exposed individuals.	60 individuals: 47 exposed to pesticides and 13 healthy individuals.

IL-6—Interleukin-6; IL-10—Interleukin-10; IL-12—Interleukin-12; TNF-γ—Tumor Necrosis Factor γ; CRP—C-Reactive Protein; CAR—CRP Serum Albumin Ratio.

**Table 4:** Summarizing the impact of various dietary components on the aging immune system and microbiota.

Dietary Component	Main Mechanism	Effect on Gut Microbiota	Effect on Immune System/Aging	References
**Curcumin**	Binds to TLRs; inhibits NF-κB and MAPK; activates PPARγ	↑ Diversity of beneficial strains (Lactobacillus and Bifidobacterium)	↓ IL-6, TNF-α, and CRP; ↑ IL-4, and IL-17; reduces inflammation	[44,45,46]
**Butyrate**	SCFA; activates GPR43/GPR109A; inhibits NF-κB and mTOR	↑ Lachnospiraceae, *Butyricicoccus*; improves intestinal barrier	↓ IL-6 and TNF-α; ↑ IL-10 and IL-18; prolongs remission in IBD	[51,52,53]
**Vitamin D3**	Regulates Th1/Th2 balance; supports Treg; inhibits IL-6/TNF-α	↓ Ruminococcus gnavus; maintains microbial stability	↓ Calprotectin; ↓ pro-inflammatory cytokines; supports mucosal immunity	[61,62]
**Omega-3 Fatty Acids**	Anti-inflammatory; modulates lipid metabolism; affects SCFA production	↑ *Coprococcus*, Bacteroides; ↓ Desulfobacterota, Klebsiella	↓ CRP and HbA1c; ↓ IL-6; improved metabolic and immune response	[67,68]
**Pesticide Exposure (Conventional Foods)**	Induces oxidative stress; damages tight junctions; activates NLRP3 inflammasome	↓ Diversity; ↑ Pathogenic species; increased permeability (“leaky gut”)	↑ IL-1β, IL-6, TNF-α, and IFN-γ; ↑ CRP and MCP-1; accelerates immunosenescence	[82,83]

TLR—Toll-Like Receptor, NF-κB—Nuclear Factor kappa-light-chain-enhancer of activated B cells, MAPK—Mitogen-Activated Protein Kinase, PPARγ—Peroxisome Proliferator-Activated Receptor Gamma, IL-1β—Interleukin 1 Beta, IL-4—Interleukin 4, IL-6—Interleukin 6, IL-10—Interleukin 10, IL-17—Interleukin 17, IL-18—Interleukin 18, TNF-α—Tumor Necrosis Factor Alpha, CRP—C-Reactive Protein, Treg—Regulatory T Cells, Th1—T Helper Type 1 Lymphocytes, Th2—T Helper Type 2 Lymphocytes, SCFA—Short-Chain Fatty Acid, GPR43—G Protein-Coupled Receptor 43, GPR109A—G Protein-Coupled Receptor 109A, mTOR—Mechanistic Target of Rapamycin, IBD—Inflammatory Bowel Disease, IFN-γ—Interferon Gamma, HbA1c—Glycated Hemoglobin, NLRP3—NLR Family Pyrin Domain-Containing 3, MCP-1—Monocyte Chemoattractant Protein 1, ↑—increase; ↓—decrease.
